# Outcomes and costs in splenectomy after failed splenic arterial embolization for blunt splenic injury^☆^

**DOI:** 10.1016/j.sopen.2025.06.011

**Published:** 2025-07-02

**Authors:** Nam Yong Cho, Bill Kwon, Esteban Aguayo, Zeyu Liu, Areti Tillou, Peyman Benharash

**Affiliations:** a Center for Advanced Surgical and Interventional Technology (CASIT), David Geffen School of Medicine, University of California, Los Angeles, CA, United States of America

**Keywords:** Splenectomy, Splenic artery embolization, Splenic salvage, Failed embolization, Spleen, Blunt trauma, Readmission

## Abstract

**Background:**

Splenic injury (BSI) is present in nearly 45 % of abdominal blunt trauma cases in the US and splenic artery embolization (SAE) has been increasingly utilized to manage BSI in recent years. However, SAE failure necessitating delayed splenectomy remains a critical concern with significant implications for patient outcomes and healthcare resource utilization.

**Methods:**

We conducted a retrospective cohort study utilizing the 2016–2021 Nationwide Readmissions Database. Adult patients (≥18 years) with BSI undergoing SAE or splenectomy were included. Early embolization was defined as SAE within 48 h of admission. Failure of SAE (FE) was defined as splenectomy following unsuccessful SAE during the index hospitalization or within 30 days post-discharge. Multivariable regression models were developed to assess the association of FE with in-hospital mortality, length of stay (LOS), and costs.

**Results:**

Of 44,750 included patients, 17,921 (40.0 %) underwent SAE as an initial operative approach. Rates of failed embolization remained stable over the study period (2016: 8.1 % vs 2021: 9.4 %, nptrend = 0.86), as did mortality following FE (2016: 1.9 % vs 2021: 1.3 %, nptrend = 0.05). After risk adjustment, early embolization was associated with reduced odds of FE (AOR 0.78, 95%CI 0.64–0.95). FE was associated with significantly increased odds of mortality (AOR 2.52, 95 % CI 1.86–3.41), prolonged LOS by 4.8 days (95 % CI 4.0–5.5), and increased hospitalization costs by $27,600 (95 % CI $24,400-30,900).

**Conclusions:**

Despite growing SAE utilization, its failure rate remains stable with FE being associated with inferior clinical and financial outcomes. Improve patient selection, increased availability of embolization and providing early embolization in select cases may enhance SAE outcomes.

## Highlights

• The embolization-first approach for blunt splenic injury has increased from 2016 to 2021.
• Embolization failure rate has remained steady over time.
• Failed embolization was associated with increased odds of mortality
• Failed embolization was associated with incremental hospital duration of stay and costs.
• High institutional embolization caseloads were associated with early embolization.

## Graphical abstract

**Figure:**
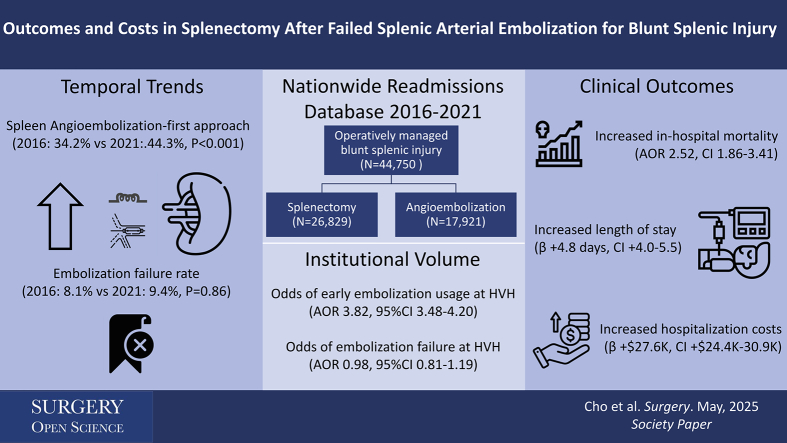

## Introduction

Splenic injury (BSI) is noted in nearly 45 % of all abdominal blunt trauma cases, resulting in approximately 40,000 hospitalizations annually with a mortality rate of up to 6.1 % [[1], [2], [3]]. Historically, severe splenic trauma was predominantly managed with surgical exploration and splenectomy. However, the introduction of nonoperative management (NOM) strategies, alongside advances in interventional radiology techniques, has shifted this care paradigm. The observed benefits of preserving functional splenic tissue and avoiding surgical risks for lower-grade injuries have prompted interest in expanding the use of splenic artery embolization (SAE) for hemodynamically stable patients with higher-grade injuries over the past few decades [[4], [5], [6]].

Despite its growing adoption, several adverse events have raised concern over the use of splenic salvage [7]. Prior studies have reported failure rates of NOM following SAE ranging from 5 to 20 %, commonly attributed to persistent bleeding, splenic infarction complicated by abscess formation, and pseudoaneurysm/rupture [8]. Moreover, recent work has demonstrated patients requiring conversion to splenectomy after initial SAE experience higher rates of postoperative complications, prolonged hospital stay, and increased resource utilization [9]. Although understanding the outcomes associated with SAE failures may inform improved BSI management, comprehensive analyses examining the trends and outcomes associated with failed SAE, remain sparse in the literature.

In the present study, we evaluated the temporal trends and outcomes of patients with BSI requiring delayed conversion to splenectomy after failed SAE. We hypothesized splenectomy after failed embolization to be associated with increased mortality, duration of stay and episodic costs.

## Methods

We performed a retrospective cohort study utilizing the 2016 to 2021 Nationwide Readmissions Database (NRD). The NRD is the largest all-payer readmissions database and is maintained as part of the Healthcare Cost and Utilization Project [10]. The NRD provides accurate estimates for ~60 % of annual hospitalizations in the United States. In addition, readmissions can be tracked across hospitals within a calendar year using unique hospital and patient-specific linkage numbers.

**Fig. 1:**
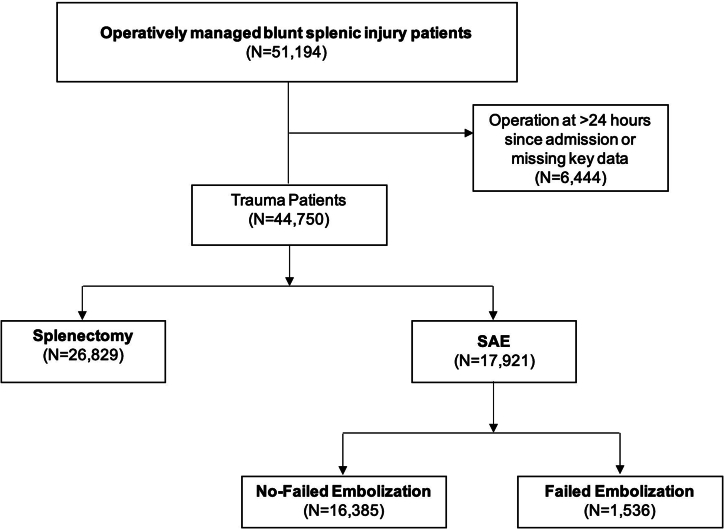
Flow chart of patients undergoing splenic arterial embolization (SAE) for blunt splenic injury.

Study selection is depicted in Fig. 1. All adult (≥18 years) hospitalizations entailing splenic arterial embolization or splenectomy with a diagnosis of BSI at index hospitalization were included using relevant *International Classification of Diseases* codes. Patients undergoing splenectomy or SAE beyond 7 days post-admission (1.2 %) were excluded to ensure analysis focused on urgent management and the acute phase of BSI. Records missing data for age, sex, cost, and in-hospital mortality were additionally excluded (0.3 %; Fig. 1). Isolated splenic injury was defined as parenchymal or vascular splenic injury, while multivisceral injury was defined as splenic injury with concomitant injuries to the head, abdomen, chest, or pelvic viscera. Early SAE was defined as occurring within 48 h of admission, while late SAE was defined as occurring between days 2 and 7 of hospitalization. Patients undergoing SAE who required subsequent splenectomy during the index hospitalization or readmission within 30 days of index discharge were grouped into the Failed Embolization (FE) cohort (Others: non-FE).

The institutional annual caseload of SAE was used to define high- and low-SAE volume tertile. Trauma Mortality Prediction Model (TMPM) was utilized to quantify the severity of injury [11]. The NRD data dictionary was used to define additional demographic and hospital characteristics [10]. The van Walraven modification of the Elixhauser Comorbidity Index was used to quantify the burden of chronic conditions [12]. Hospitalization costs were calculated by application of hospital-specific cost-to-charge ratios and subsequently inflation-adjusted to the 2021 Personal Health Care Expenditure Index [13]. Perioperative complications included cardiovascular (cardiac arrest, myocardial infarction, tamponade), respiratory (acute respiratory failure, prolonged ventilation ≥96 h, pneumonia), gastrointestinal (bowel ischemia, hemoperitoneum), thromboembolic (deep venous thrombosis, pulmonary embolism) and infectious (sepsis, surgical site infection) sequelae. The primary outcome of interest was in-hospital mortality, while secondary outcomes included perioperative complications, hospitalization duration of stay (LOS), hospitalization costs, non-home discharge, and 30-day readmission.

Categorical variables are reported as percentages (%), while continuous variables are reported as medians with an interquartile range (IQR). We used Pearson's χ2 and Mann-Whitney *U* tests to compare categorical and continuous variables, respectively. Cuzick's non-parametric rank-based test (nptrend) was utilized to evaluate the significance of temporal trends [14]. Entropy balancing was employed to obtain a more homogenous distribution of covariates between the FE and non-FE groups. Of note, entropy balancing maintains the entire cohort and generates comparable groups utilizing a reweighting algorithm analysis [15]. Multivariable regression models were then applied to evaluate the association between FE and the outcomes of interest. Adjusted outcomes are reported as adjusted odds ratio (AOR) or beta coefficients (β) for dichotomous and continuous variables with a 95 % confidence interval (CI). An α < 0.05 was set for statistical significance. All statistical analyses were performed using Stata 16.0 (StataCorp, College Station, TX). Due to the deidentified nature of the NRD, this study was deemed exempt from full review by the Institutional Review Board at the University of California, Los Angeles.

## Results

### Demographic data and unadjusted outcomes

**Fig. 2:**
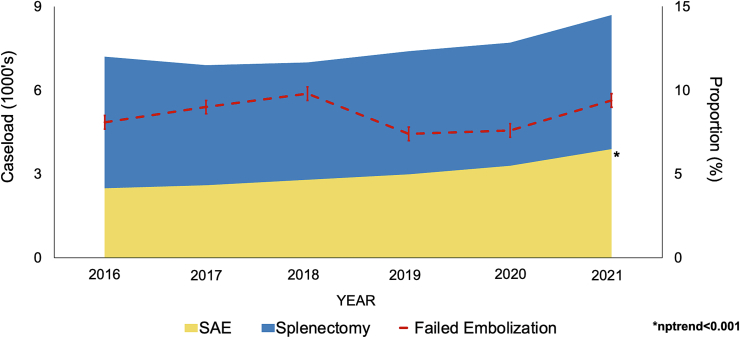
Temporal trends in injury severity estimated by Trauma Mortality Prediction Model (TMPM) in patients undergoing splenic arterial embolization for blunt splenic injury, stratified by failed embolization (FE) and successful embolization (Non-FE).

**Fig. 3:**
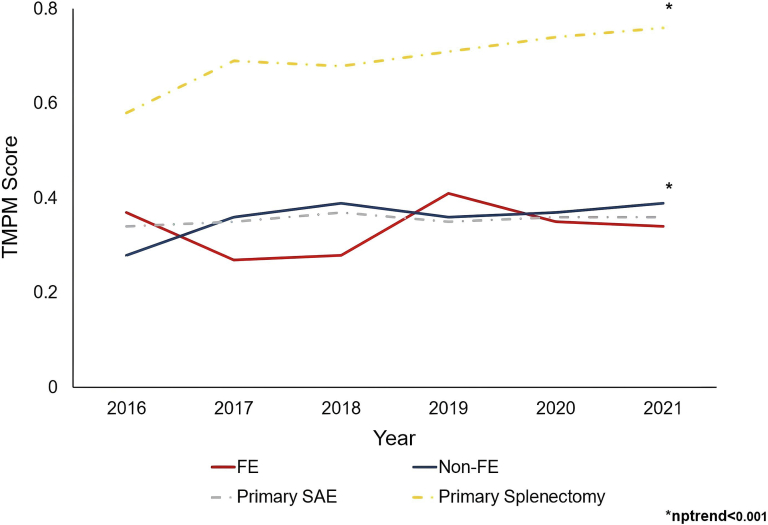
Temporal trends in utilization of splenic arterial embolization and splenectomy as the primary operative approach for blunt splenic injury management as well as proportion of failed spleen embolization.

Of an estimated 44,750 patients meeting the inclusion criteria, 17,921 (40.0 %) underwent SAE as the initial approach, while 26,829 (60.0 %) had primary splenectomy. Over the study period, the utilization of SAE increased from 34.2 % to 44.3 % (nptrend<0.001). Rates of failed embolization remained stable over the study period (2016: 8.1 % vs 2021: 9.4 %, nptrend = 0.86), as did mortality following FE (2016: 1.9 % vs 2021: 1.3 %, nptrend = 0.05) (Fig. 2). Injury severity measured by TMPM remained steady for FE (2016: 0.37 [0.06–0.77] vs 2021: 0.34 [0.08–0.77], nptrend = 0.09) while injury severity increased in the non-FE cohort over the study period (Table 1; Fig. 3).

**Table 1:** Demographic information in patients undergoing SAE stratified by failed embolization (FE) status, which was defined as conversion to splenectomy during index hospitalization or at readmission. TMPM, Trauma Mortality Prediction Model.

	Non-FE (N = 16,385)	FE (N = 1536)	P-value
Age (year)	45 [30–61]	49 [34–61]	0.002
Female sex (%)	32.2	32.2	0.98
Elixhauser Index Score	1 [0–2]	2 [1–3]	<0.001
TMPM	0.38 [0.08–0.77]	0.34 [0.06–0.78]	0.10
Insurance status (%)			0.17
Medicare	16.9	18.7	
Medicaid	20.4	24.0	
Private	43.7	40.0	
Self-pay	9.5	9.2	
Other	9.5	8.1	
Hospital bed size (%)			0.07
Small	4.9	6.6	
Medium	21.1	23.5	
Large	74.0	69.9	
Hospital ownership (%)		0.47	
Government	17.2	18.7	
Nonprofit	73.3	71.0	
For-profit	9.5	10.3	
Chronic anemia (%)	1.8	0.9	0.06
Bowel ischemia (%)	0.2	1.9	<0.001
Coronary artery disease (%)	6.2	6.7	0.57
Coagulopathy (%)	8.9	17.2	<0.001
Diabetes (%)	9.8	9.3	0.67
ESRD (%)	1.2	1.3	0.8
Heart failure (%)	4.3	6.1	0.02
Hemoperitoneum (%)	13.2	22.1	<0.001
Hypertension (%)	4.8	6.4	0.06
Liver disease (%)	5.7	11.4	<0.001
Smoking (%)	22.9	20.5	0.19

On unadjusted analysis, FE patients experienced a higher rate of inpatient mortality compared to non-FE (8.4 vs 3.4 %, P < 0.001) (Table 2; Fig. 3). Over the study period, mortality among FE remained stable (2016: 7.8 vs 2020: 9.3 %, P = 0.24). Additionally, those in the FE cohort had higher rates of cardiac (4.3 vs 1.5 %), gastrointestinal (24.3 vs 13.5 %, P < 0.001), respiratory (24.1 vs 12.2 %), and infectious complications (11.2 vs 3.1 %; all P < 0.001) (Table 2). Compared to others, FE patients had longer LOS (10 [6–17] vs 5 [3–10] days, P < 0.001) and greater index hospitalization costs ($57,100 [$33,900-90,600] vs $33,000 [$22,600-53,200], P < 0.001). Patients in the FE cohort had greater rates of non-home discharge (34.9 vs 27.4 %, P < 0.001) and 30-day readmission (23.7 vs 8.2 %, P < 0.001).

**Table 2:** Bivariate comparison of clinical and financial outcomes in patients undergoing SAE stratified by failed embolization (FE) status. D, days.

	Non-FE (N = 16,386)	FE (N = 1536)	P-value
Early embolization (%)	84.8	80.2	0.004
Mortality (%)	3.4	8.4	<0.001
Cardiac complication (%)	1.5	4.3	<0.001
Gastrointestinal complication (%)	13.5	24.3	<0.001
Respiratory complication (%)	12.2	24.1	<0.001
Infectious complication (%)	3.1	11.2	<0.001
Thromboembolic complication (%)	1.6	2.6	0.02
Length of stay (d)	5 [3–10]	10 [6–17]	<0.001
Cost ($1000)	33.0 [22.6–53.2]	57.1 [33.9–90.6]	<0.001
Nonhome discharge (%)	27.4	34.9	<0.001
Readmission < 30 days (%)	8.2	23.7	<0.001

### Adjusted outcomes of FE with non-FE as reference

After adequate risk adjustment and with non-FE as a reference, early embolization was associated with reduced odds of FE (AOR 0.78, 95%CI 0.64–0.95). FE was associated with increased odds of mortality (AOR 2.52, 95%CI 1.86–3.41), as well as cardiac, respiratory, and thromboembolic complications (Table 3). FE s was associated with increased odds of gastrointestinal (AOR 2.93, 95%CI 1.98–4.33) and infectious (AOR 4.07, 95%CI 3.11–5.33) complications. Furthermore, compared to non-FE, FE status was linked to longer LOS by 4.8 days (95%CI 4.0–5.5 days), higher incremental index hospitalization costs by $27,600 (95%CI $24,400-30,900), and higher 30-day readmission (AOR 3.32, 95%CI 2.74–4.02) (Fig. 3).

**Table 3:** Risk adjusted outcomes of patients with failed embolization (FE) status compared to those without (Non-FE). Estimates are reported with adjusted odds ratio and beta coefficient with 95 % confidence interval (CI) for logistic and linear regression outputs, respectively. D, days.

	FE (ref: Non-FE)	
	Estimates	95 % CI
Early embolization	0.78	0.64–0.95
Mortality	2.52	1.86–3.41
Cardiac complication	2.92	1.98–4.33
Gastrointestinal complication	2.93	1.98–4.33
Respiratory complication	2.38	1.96–2.89
Infectious complication	4.07	3.11–5.33
Thromboembolic complication	1.96	1.25–3.07
Length of stay (d)	4.77	3.99–5.54
Cost ($1000)	27.6	24.4–30.9
Nonhome discharge	1.46	1.22–1.75
30-day readmission	3.32	2.74–4.02

### Volume tertile analysis

Hospitals in the low-volume tertile of embolization performed lower annual SAE compared to high-volume centers (2 [1–3] cases vs 14 [12–21] cases, P < 0.001). Compared to those managed at LVH, patients at HVH were younger (39 [26–57] vs 44 [29–60] years, P < 0.001) and more commonly had severe injuries (0.73 [0.20–0.83] vs 0.41 [0.07–0.80], P < 0.001). Following risk adjustment, management at HVH was associated with similar odds of embolization failure (AOR 0.98, 95%CI 0.81–1.19) while having increased odds of early embolization usage (AOR 3.82, 95%CI 3.48–4.20). HVH management was associated with reduced odds of in-hospital mortality (AOR 0.82, 95%CI 0.71–0.96) compared to LVH (Table 4). Moreover, management at HVH was associated with lower odds of cardiac (AOR 0.71, 95%CI 0.64–0.80) and respiratory (AOR 0.64, 95%CI 0.58–0.71) complications. Management at HVH was significantly associated with reduced LOS by 0.8 days (95%CI 0.2–1.4 days) and lower odds of non-home discharge (AOR 0.82, 95%CI 0.76–0.90). HVH status was not associated with incremental hospitalization costs or odds of 30-day readmission.

**Table 4:** Risk adjusted outcomes of patients undergoing splenic arterial embolization (SAE) for blunt splenic injury at high SAE volume centers (HVH) with the management at low-volume hospitals (LVH) as reference. Estimates are reported with adjusted odds ratio and beta coefficient with 95 % confidence interval (CI) for logistic and linear regression outputs, respectively. D, days.

	HVH (ref: LVH)	
	Estimates	95 % CI
Embolization failure	0.98	0.81–1.19
Early embolization	3.82	3.48–4.20
Mortality	0.82	0.71–0.96
Cardiac complication	0.71	0.64–0.80
Gastrointestinal complication	0.66	0.60–0.73
Respiratory complication	0.64	0.58–0.71
Infectious complication	1.95	0.81–1.11
Thromboembolic complication	0.92	0.71–1.19
Length of stay (d)	−0.8	−1.4–0.2
Cost ($1000)	−1.2	−3.8–1.5
Nonhome discharge	0.75	0.68–0.83
30-day readmission	1.03	0.89–1.19

## Discussion

The decision to preserve the spleen in trauma management necessitates a careful balance of clinical factors and evidence-based protocols to optimize patient outcomes and resource allocation. While SAE has emerged as a widely adopted spleen-preserving intervention for blunt splenic injury, data on its failure rates remain limited. In the present study, despite the increased utilization of SAE over the study period, rates of splenectomy following unsuccessful embolization, and its associated inpatient mortality remained steady. Early embolization was associated with reduced odds of embolization failure. Additionally, we demonstrated splenectomy after failed SAE to be associated with increased odds of mortality and greater resource utilization. Moreover, our findings suggest that institutional SAE caseload did not influence the success of SAE, although mortality following its failure was lower at high-volume hospitals. Given the implications of refining protocols and improving clinical outcomes in patients undergoing SAE for BSI, our findings warrant further discussion.

Given the well-documented increased lifetime risks of thromboembolism and infectious complications associated with splenectomy, the importance of spleen preservation cannot be overstated [16]. Consequently, trauma teams and patients prioritize spleen-preserving strategies whenever clinically feasible. In the present study, the increasing injury severity observed among patients with successful embolization suggests a growing proficiency among trauma teams in managing high-grade splenic injuries with NOM. Indeed, prior work has shown NOM to be just as effective as immediate splenectomy for hemodynamically stable adult patients with grade IV and V blunt splenic injuries [17]. Despite such improvement in NOM and increased SAE utilization, challenges remain in optimizing its success. In the present study, we showed that 8.5 % of patients undergoing SAE have had a conversion to splenectomy in the index hospitalization or 30-day readmission. Additionally, rates of conversion to splenectomy, as well as mortality following failed embolization, have remained stable over the study period. In patients with low acuity injuries amenable to NOM, patients have comparable outcomes following prophylactic SAE or surveillance and then embolization only if necessary [18]. However, in patients with more severe BSI necessitating SAE within the first 24 h of admission, the decision to proceed with splenectomy presents a complex and nuanced dilemma.

Current guidelines from the World Society of Emergency Surgery (WSES) guideline recommend laparotomy and splenectomy in cases of angioembolization [18]. Yet, substantial variability exists across trauma centers regarding embolization techniques and protocols regarding postprocedural monitoring, especially among those who are hemodynamically stable [19,20]. Such a lack of standardized protocols hinders improvement in embolization success rate and mortality. In the present study, we showed early embolization to be associated with reduced odds of embolization failure. Identifying factors for early surgical interventions, such as shock index >0.9, grade V splenic injury and increased transfusion requirements within 24 h of admission, may optimize patient outcomes [21]. Additionally, Minor to moderate splenic trauma is currently recommended for nonoperative management with consideration for an angiogram if a positive blush is found in the contrast study [18]. While contrast blush or pseudoaneurysm is currently the most common indication for SAE, prior work has shown extravasation from the spleen at the time of admission to be associated with greater odds of delayed splenectomy [3,7]. Given our findings regarding HVH having a 3-fold likelihood of providing early embolization compared to LVH, it is likely that NOM protocols with adjunct usage of SAE are stringent upon available resources and intuitional protocols. A proactive decision to investigate and monitor contrast extravasation as well as providing timely SAE may enhance embolization success and improve patient survival.

In recent years, angioembolization has been shown to have a comparable outcome in terms of mortality [22,23]. However, our findings demonstrated that patients undergoing splenectomy following unsuccessful embolization face significantly increased odds of mortality and perioperative complications. Despite stable embolization failure rates over time, failed SAE continues to pose a substantial risk to trauma patients with BSI. Our findings demonstrated that conversion to splenectomy in patients with failed SAE was associated with an additional 4.8 days of hospital stay and increased hospitalization costs of $27,600. These resource burdens are nearly double that of initial SAE or planned splenectomy. Given the median trauma patient LOS of three days, extended stay by nearly 5 days significantly strains trauma center capacity [24]. Improved patient selection and protocols to provide SAE, as well as judicious use of primary splenectomy, may improve patient outcomes and resource allocation in trauma care.

Across various surgical disciplines, higher operative volume at institutions has been associated with improved patient outcomes, including lower complication and mortality rates [25]. Nonetheless, previous studies have demonstrated that the overall success rate of NOM and SAE failure rates are comparable between high- and low-volume centers [26,27]. Similarly, we showed that institutional SAE volume was not associated with a higher SAE success rate. However, we found that management at HVH was associated with significantly reduced odds of mortality and perioperative complications among patients undergoing the SAE-first approach for BSI. Of note, the current recommendation from the American College of Surgeons requires interventional radiology response for hemorrhage control to be available at Level I and II trauma centers [28]. Given most HVH are level I or II trauma centers, improved outcomes observed at HVHs are likely attributable to the presence of multidisciplinary teams capable of effectively managing splenic injury progression [29]. Notably, our findings showed that management at HVH was also associated with reduced resource utilization, including shorter LOS, lower hospitalization costs, and decreased rates of non-home discharge. The enhanced expertise of surgeons and the implementation of streamlined protocols at HVHs likely contribute to improved operating room efficiency, which may optimize financial performance [30,31]. Systemic efforts to expand the accessibility and adoption of angioembolization at low-volume trauma centers may allow for broader availability of spleen-preserving treatment options and improve SAE success rates.

The present study had several important limitations. Due to the retrospective nature of the database, we could not assess causality. Although we adjusted for the severity of splenic injury by using the injury severity metric and focusing on operative-managed blunt splenic injury, our analysis of splenic injury grades was limited. We could not assess imaging data, which could have influenced the management modalities in patients. The exact timings of embolization or the initiation of interventional radiology response could not be determined. While we looked at those who failed embolization and had a subsequent splenectomy, we could not evaluate patients who have had multiple re-embolization. We also could not assess regional or institutional trauma system differences or the American College of Surgeon trauma center level verification.

In conclusion, optimizing the management of blunt splenic injury requires a delicate balance between spleen preservation and timely surgical intervention. Our findings highlight the increasing utilization of SAE in recent years and its potential to manage high-grade splenic injuries effectively. However, the persistent rates and risks associated with failed embolization—including increased mortality, prolonged hospital stays, and greater resource utilization—underscore the need for refined institutional protocols. Expanding access to early embolization, improving patient selection criteria, and enhancing interventional radiology capabilities at lower-volume centers may improve BSI management and patient outcomes.

## CRediT authorship contribution statement

**Nam Yong Cho:** Conceptualization, Investigation, Methodology, Validation, Visualization, Writing – original draft, Writing – review & editing. **Bill Kwon:** Conceptualization, Methodology, Writing – review & editing. **Esteban Aguayo:** Methodology, Validation, Visualization, Writing – review & editing. **Zeyu Liu:** Writing – original draft. **Areti Tillou:** Conceptualization, Validation. **Peyman Benharash:** Conceptualization, Methodology, Supervision, Validation, Writing – review & editing.

## Ethics approval

Authors followed ethics guidelines provided by Elsevier.

## Sources of funding

None.

## Declaration of competing interest

The authors report no proprietary or commercial interest in any product mentioned or concept discussed in this manuscript.

## Appendix A Supplementary data

Supplementary tables Image 1
