# Supplementary material for: Outcomes and costs in splenectomy after failed splenic arterial embolization for blunt splenic injury
Source: Surg Open Sci. 2025 Jul 2;27:61–7. doi: 10.1016/j.sopen.2025.06.011 (PMC12274320; doi:10.1016/j.sopen.2025.06.011)
Supplement: Supplementary file 1 — Supplementary tables [file mmc1.docx]

Supplemental Table 1. Demographic information in patients undergoing primary SAE or splenectomy for blunt splenic injury.

|  | **SAE**  **(N=17,921)** | **Splenectomy (N=26,829)** | **P-value** |
| --- | --- | --- | --- |
| Age (year) | 46 [31-61] | 42 [29-59] | <0.001 |
| Female Sex (%) | 32.2 | 30.3 | 0.005 |
| Elixhauser Index Score | 1 [0-2] | 1 [0-2] | 0.22 |
| Insurance Status (%) |  |  | <0.001 |
| Medicare | 17.0 | 16.1 |  |
| Medicaid | 20.7 | 25.8 |  |
| Private | 43.3 | 37.0 |  |
| Self-Pay | 9.5 | 11.9 |  |
| Other | 9.5 | 9.2 |  |
| Hospital Bed Size (%) |  |  | <0.001 |
| Small | 5.0 | 7.2 |  |
| Medium | 21.4 | 21.8 |  |
| Large | 73.6 | 71.0 |  |
| Hospital Ownership (%) |  |  | <0.001 |
| Government | 17.4 | 22.8 |  |
| Nonprofit | 73.1 | 67.2 |  |
| For-profit | 9.5 | 10.0 |  |
| Anemia (%) | 1.7 | 1.6 | 0.8 |
| Bowel Ischemia (%) | 0.4 | 1.1 | <0.001 |
| Coronary Artery Disease (%) | 6.2 | 5.3 | 0.01 |
| Coagulopathy (%) | 9.7 | 15.4 | <0.001 |
| Diabetes (%) | 9.7 | 6.5 | <0.001 |
| ESRD (%) | 1.9 | 0.9 | 0.02 |
| Heart Failure (%) | 4.5 | 3.6 | 0.002 |
| Hemoperitoneum (%) | 14.0 | 17.5 | <0.001 |
| Hypertension (%) | 5.0 | 3.7 | <0.001 |
| Liver Disease (%) | 6.2 | 6.3 | 0.81 |
| Smoking (%) | 22.6 | 16.1 | <0.001 |

Supplemental Table 2. Bivariate comparison of clinical and financial outcomes in patients undergoing primary splenic arterial embolization (SAE) or splenectomy for blunt splenic injury. D, days.

|  | SAE  (N=17,921) | Splenectomy (N=26,829) | P-Value |
| --- | --- | --- | --- |
| Mortality (%) | 3.8 | 12.3 | <0.001 |
| Cardiac Complication (%) | 1.7 | 6.8 | <0.001 |
| Gastrointestinal Complication (%) | 14.4 | 19.1 | <0.001 |
| Respiratory Complication (%) | 13.2 | 29.3 | <0.001 |
| Infectious Complication (%) | 3.8 | 9.5 | <0.001 |
| Thromboembolic Complication (%) | 1.7 | 3.0 | <0.001 |
| Length of Stay (D) | 6 [3-11] | 9 [5-17] | <0.001 |
| Cost ($1,000) | 34.3 [23.2-57.1] | 43.2 [24.5-85.2] | <0.001 |
| Nonhome Discharge (%) | 28.1 | 36.3 | <0.001 |
| Readmission < 30 Days (%) | 9.5 | 9.2 | 0.44 |

Supplemental Table 3. Risk adjusted outcomes of patients undergoing primary splenectomy compared to those undergoing primary splenic arterial embolization (SAE) for blunt splenic injury. Estimates are reported with adjusted odds ratio and beta coefficient with 95% confidence interval (CI) for logistic and linear regression outputs, respectively. D, days.

|  | Splenectomy (Ref: SAE) | | |  |
| --- | --- | --- | --- | --- |
|  | | Estimates | 95%CI | |
| Mortality | | 3.01 | 2.70-3.51 | |
| Cardiac Complication | | 3.28 | 2.76-3.91 | |
| Gastrointestinal Complications | | 3.37 | 2.83-4.01 | |
| Respiratory Complication | | 2.34 | 2.16-2.52 | |
| Infectious Complication | | 2.45 | 2.15-2.79 | |
| Thromboembolic Complication | | 1.62 | 1.33-1.96 | |
| Length of Stay (d) | | 3.26 | 2.86-3.66 | |
| Cost ($1,000) | | 12.5 | 10.7-14.3 | |
| Nonhome Discharge | | 1.38 | 1.29-1.48 | |
| 30-Day Readmission | | 0.99 | 0.90-1.09 | |

Supplemental Table 4. Demographic information in patients undergoing SAE stratified by management at high- (HVH) and low-volume hospitals (LVH), which was defined as conversion to splenectomy during index hospitalization or at readmission. TMPM, Trauma Mortality Prediction Model.

|  | HVH  (N=5,680) | LVH  (N=6,799) | P-Value |
| --- | --- | --- | --- |
| Age (year) | 39 [26-57] | 44 [29-60] | <0.001 |
| Female Sex (%) | 30.2 | 30.8 | 0.46 |
| Elixhauser Index Score | 1 [0-2] | 1 [0-2] | 0.21 |
| TMPM | 0.73 [0.20-0.83] | 0.41 [0.07-0.80] | <0.001 |
| Insurance Status (%) |  |  | <0.001 |
| Medicare | 12.6 | 17.8 |  |
| Medicaid | 24.3 | 25.0 |  |
| Private | 42.2 | 38.1 |  |
| Self-Pay | 11.2 | 10.3 |  |
| Other | 9.7 | 8.8 |  |
| Hospital Bed Size (%) |  |  | <0.001 |
| Small | 0.2 | 10.3 |  |
| Medium | 15.4 | 26.0 |  |
| Large | 84.4 | 63.7 |  |
| Hospital Ownership (%) |  |  | <0.001 |
| Government | 21.3 | 18.8 |  |
| Nonprofit | 75.2 | 69.0 |  |
| For-profit | 3.4 | 12.2 |  |
| Anemia (%) | 0.7 | 2.2 | <0.001 |
| Bowel Ischemia (%) | 0.9 | 0.7 | 0.22 |
| Coronary Artery Disease (%) | 4.5 | 6.2 | <0.001 |
| Coagulopathy (%) | 11.8 | 13.8 | 0.02 |
| Diabetes (%) | 6.2 | 7.9 | 0.004 |
| ESRD (%) | 0.9 | 0.9 | 0.59 |
| Heart Failure (%) | 2.8 | 4.3 | <0.001 |
| Hemoperitoneum (%) | 12.0 | 18.9 | <0.001 |
| Hypertension (%) | 3.3 | 4.7 | <0.001 |
| Liver Disease (%) | 5.7 | 6.3 | 0.30 |
| Smoking (%) | 17.1 | 18.5 | 0.10 |

Supplemental Table 5. Bivariate comparison of clinical and financial outcomes in patients undergoing SAE stratified by management at high- and low-volume hospitals. D, days.

|  | HVH  (N=5,680) | LVH  (N=6,799) | P-Value |
| --- | --- | --- | --- |
| Embolization Failure (%) | 8.0 | 9.6 | 0.14 |
| Mortality (%) | 8.9 | 9.2 | 0.64 |
| Cardiac Complication (%) | 4.5 | 5.2 | 0.09 |
| Gastrointestinal Complication (%) | 13.2 | 18.6 | <0.001 |
| Respiratory Complication (%) | 21.5 | 25.4 | 0.002 |
| Infectious Complication (%) | 7.7 | 7.3 | 0.48 |
| Thromboembolic Complication (%) | 2.7 | 2.2 | 0.12 |
| Length of Stay (d) | 7 [4-14] | 7 [4-15] | 0.06 |
| Cost ($1,000) | 39.7 [24.5-76.1] | 37.4 [23.2-68.8] | 0.07 |
| Nonhome Discharge (%) | 30.8 | 32.4 | 0.21 |
| Readmission < 30 Days (%) | 9.2 | 8.4 | 0.18 |
